# Environmental DNA sequencing reveals the regional difference in diversity and community assembly mechanisms of eukaryotic plankton in coastal waters

**DOI:** 10.3389/fmicb.2023.1132925

**Published:** 2023-02-10

**Authors:** Zhen-Guang Yan, Xue-Ming Zhu, Shou-Wen Zhang, Hua Jiang, Shu-Ping Wang, Chao Wei, Jie Wang, Yun Shao, Chen Liu, Hui Wang

**Affiliations:** ^1^State Key Laboratory of Environmental Criteria and Risk Assessment, Chinese Research Academy of Environmental Sciences, Beijing, China; ^2^Frontiers Research Center, Southern Marine Science and Engineering Guangdong Laboratory, Zhuhai, China; ^3^Marine Climate Prediction and Assessment Center, National Marine Environmental Forecasting Center, Beijing, China

**Keywords:** environmental DNA, eukaryotic plankton, biodiversity, community assembly, coastal waters

## Abstract

The diversity and community assembly mechanisms of eukaryotic plankton in coastal waters is so far not clear. In this study, we selected the coastal waters of Guangdong-Hong Kong-Macao Greater Bay Area, which is a highly developed region in China, as the research area. By use of high-throughput sequencing technologies, the diversity and community assembly mechanisms of eukaryotic marine plankton were studied in which a total of 7,295 OTUs were obtained, and 2,307 species were annotated by doing environmental DNA survey of 17 sites consist of surface and bottom layer. Ultimately, the analysis reveals that the species abundance of bottom layer is, by and large, higher than that in the surface layer. In the bottom, Arthropoda is the first largest group, accounting for more than 20% while Arthropoda and Bacillariophyta are dominant groups in surface waters accounting for more than 40%. It is significant of the variance in alpha-diversity between sampling sites, and the difference of alpha-diversity between bottom sites is greater than that of surface sites. The result suggests that the environmental factors that have significant influence on alpha-diversity are total alkalinity and offshore distance for surface sites, and water depth and turbidity for bottom sites. Likewise, the plankton communities obey the typical distance-decay pattern. Analysis about community assembly mechanisms reveals that, overall, dispersal limitation is the major pattern of community formation, which accounts for more than 83% of the community formation processes, suggesting that stochastic processes are the crucial assembly mechanism of the eukaryotic plankton community in the study area.

## 1. Introduction

Eukaryotic marine plankton form the basis of the ocean’s food-web (Benedetti et al., 2021), and have an essential role to display in energy flow and elemental cycling of the marine ecosystem (Hutchins and Fu, 2017; Vorobev et al., 2020). Spatiotemporal distribution patterns, biodiversity, and the related mechanisms are the significant research contents of ecology (Martiny et al., 2006). The diversity of marine plankton has attracted much attention (Sauterey et al., 2017; Ibarbalz et al., 2019; Selander et al., 2019; Trubovitz et al., 2020). For example, a study in the northwest Atlantic retrieved that Copepods can affect the structure of marine plankton community by releasing chemicals (Selander et al., 2019). Previous studies also indicated that variation of microeukaryotic diversity was chiefly ascertained by variations in levels of dissolved oxygen (DO), nutrients, salinity, and turbidity in a river-to-estuary gradient ecosystem (Xu et al., 2020), and alpha-diversity of benthic protists was much more significant than that of plankton in an intertidal zones, whereas no distinct patterns of organism size/seasonal distribution were observed for either community (Kong et al., 2019). Lopez-Garcia et al. (2007) carried out a molecular survey based on environmental DNA (eDNA) of eukaryotic communities in a unique off-axis hydrothermal vent field, and found eight major taxa, such as Metazoan, Fungi. In addition, river microeukaryotes exhibit in stark contrast in community well-being in both wet and dry seasons (Chen et al., 2019). However, there are still scarcely any studies about the eukaryotic plankton diversity and community assembly mechanisms in coastal waters up to now, neither is the understanding of the plankton communities in the coastal waters deep enough yet.

In recent years, community assembly mechanism has become one of the hot topics in ecology. Vellend (2010) proposed a conceptual synthesis, which divided the ecological processes of community formation into four types, namely selection, dispersal, ecological drift, and diversification. Furtherly, Stegen et al. (2013) established an analytical framework to estimate the relative importance of dispersal (dispersal limitation and homogenizing dispersal), selection (heterogeneous and homogenizing selection), and ecological drift. In terms of this mechanistic predictive framework, the relative importance of the assembly processes has been illustrated in lentic waters (Yan et al., 2017; Danczak et al., 2018), and marine ecosystems (Logares et al., 2018) by using a phylogenetic null model (Stegen et al., 2013), which enables the quantification of the influences of various ecological processes involved in the assembly of microbes. In addition, it is a fundamental element of spatial distribution patterns to clarify the biotic communities’ formation and maintenance mechanism, in which distance-decay patterns of microbial communities have been stated oftentimes in freshwater lakes and rivers (Logares et al., 2018; Isabwe et al., 2019; Liu et al., 2020), ocean (Wu et al., 2018, 2020; Kong et al., 2020), and intertidal zones (Kong et al., 2019). However, the assembly processes of community establishment and maintenance for generating regional distribution patterns such as distance-decay, are remained to clarify in eukaryotic plankton ecology.

Recently, the progressed high throughput genome sequencing technology has been one of powerful tools to obtain biological big data, laying a good foundation for the study of biodiversity and related mechanisms (Wang et al., 2019, 2021; Fan et al., 2020). By eDNA/RNA technology, not only can researchers obtain the rich information of the biological community in the environment, but also be able to further analyze the ecological process and mechanisms involved. For example, Bass and Cavalier-Smith (Bass and Cavalier-Smith, 2004) conducted the first 18S rRNA multi-library environmental PCR survey of Cercozoa from a range of different habitats, revealed remarkably high global biodiversity of Cercozoa, and proposed a new insight into its evolution and classification. In another research supported by Kong et al., the diversity distribution and assembly mechanisms of planktonic and benthic microeukaryote communities in intertidal zones was analyzed by terms of environmental RNA technology (Kong et al., 2019). On all these counts, the costal ocean is closely related to human activities, and its biodiversity and community formation mechanisms are now widely concerned. In this study, we delved into the biodiversity and assembly mechanisms of eukaryotic plankton communities directly at the coastal waters of the Guangdong-Hong Kong-Macao Greater Bay Area, China. Separately, samples were gathered from both the surface and bottom layers. To be specific, our research is committed to elucidate the following issues: (i) What are the characteristics of eukaryotic plankton community diversity in coastal waters? Is there any difference of the diversity between the surface and bottom layers? (ii) What are the factors that influence the distribution of eukaryotic plankton communities? (iii) What is the relative importance of stochastic and deterministic assembly processes of eukaryotic plankton community?

## 2. Materials and methods

### 2.1. Sample collection

**Figure 1:**
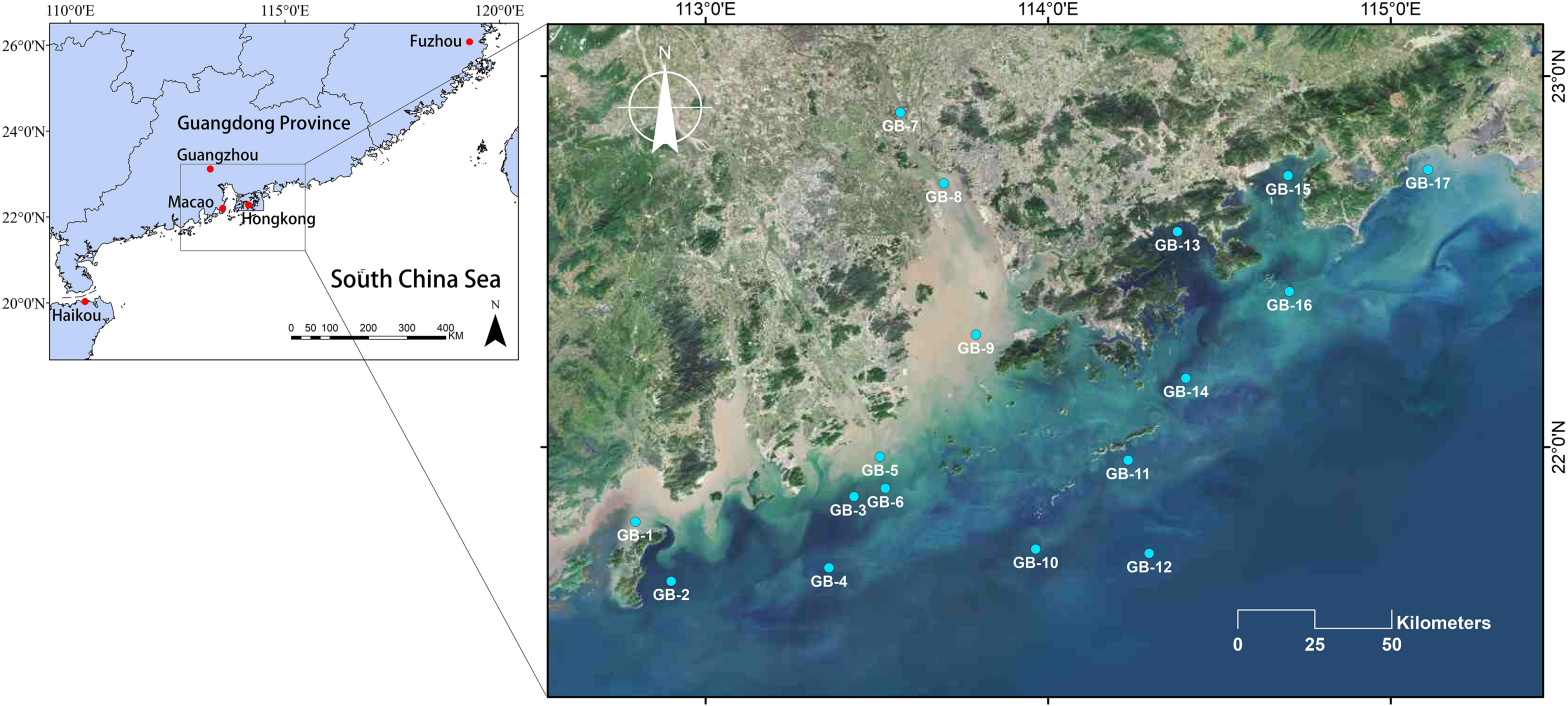
Sampling sites of the coastal waters of Guangdong-Hong Kong-Macao Greater Bay Area, China. Among these sites, GB-7, GB-8, and GB-9 are all located in the Pearl River Estuary which is the second largest estuary in China.

As is illustrated in Figure 1, this survey was conducted in coastal waters of Guangdong-Hong Kong-Macao Greater Bay Area, China, 2021, with an overall number of 17 sampling points, running from July 27 to August 9 by ship. Each of these 17 points was sampled at the surface and bottom layers, respectively. A total of 34 water samples were collected during sampling. The 17 surface water samples are numbered from GB-1S to GB-17S, while the 17 bottom water samples are numbered from GB-1B to GB-17B. During the investigation, *in situ* water quality parameters were determined using CTD, such as ORP, EC, T, salinity, depth, DO, pH, chlorophyll a, turbidity, and irradiance (E). For each sample, two liters of water were collected and divided into two subsamples, one for eukaryotic plankton community analyses and the other for water chemistry determination. According to the Specification for Marine Monitoring, Part 4: Seawater Analysis (GB17378.4-2007), nitrate, nitrite, DOC, TC, NH_4_^+^, DIC, and PO43-were assessed. In addition, total alkalinity (TALK) was determined according to the industry standard SL 83-1994. For the community analyses, 1,000 ml of water was filtered through 0.22 μm Millipore polycarbonate filters (Wang et al., 2021), and at the same time, the same volume of distilled water was filtered as the negative control. Ulteriorly the filters containing plankton were stored at −80°C for further treatment.

### 2.2. DNA extraction and sequencing

Total eDNA was extracted using the cetyl trimethylammonium bromide (CTAB) method combined with the Zymo DNA Clean & Concentrator kit (Zymo Research Corp, Irvine, United States; Fan et al., 2020). The eDNA concentration was determined using a NanoDrop spectrophotometer (ND-1000, Thermo Fisher Scientific, United States). The extracted DNA was stored at-80°C until further treatment. The V9 region of the 18S rRNA genes of eukaryotic plankton were PCR amplified using the eDNA as templates with the primer pairs 1380F (5′-CCCTGCCNTTTGTACACAC-3′) and 1510R (5′-CCTTCN GCAGGTTCACCTAC-3′; Amaral-Zettler et al., 2009; de Vargas et al., 2015). Three replicates were set in the PCR amplification experiment. An initial activation step was employed in the PCR protocol at 95°C for 5 min, followed by 30 cycles consisting of 94°C for 30 s, 57°C for 45 s, and 72°C for 1 min; and end up with a 2-min extension at 72°C. Corresponding to each sample, added the purified PCR products with 8-base sequence tags, and a MiSeq sequencing platform (Illumina, San Diego, United States) was employed for high throughput sequencing. All of the low-quality sequencing data points with adaptors, low complexity, ambiguous bases, and those equipped with average quality scores less than 20 were discarded in virtue of the UPARSE pipeline (Edgar, 2013). At the threshold of 97% sequence-similarity, the 18S rRNA gene sequences were categorized into operational taxonomic units (OTUs) using USEARCH (v11.0.667; Edgar, 2013). Taxonomic annotation analysis was performed using the Qiime2 pipeline (Caporaso et al., 2010) with respect to the database SILVA 18S^1^ and database NCBI 18S (Fan et al., 2020).^2^ The results were filtered then, and sequences that simultaneously meet both the criteria of similarity greater than 90% and coverage greater than 90% were assigned to different taxonomic levels, while those that did not meet the criteria were classified as “unclassified.” The remaining high-quality data were transformed to relative proportions before conducting subsequent statistical analysis.

### 2.3. Data analysis

All statistical analyses as well as the production of figures were executed with R project (v4.2.2; R Core Team, 2022), and the biological data was analyzed based on the OTU data at the species level unless otherwise stated. Venn diagram was drawn with R VennDiagram package (v1.6.20; Chen and Boutros, 2011) to show the differences in species composition between the surface and bottom layers of each site. The species which were with more than 1% abundance in all samples were considered as the dominant species, and the other species were considered as others to identify the dissimilarities in species community composition at different regions and sites (both in surface and bottom). Additionally, the alpha-diversity (Shannon index) of each sampling site and its correlation with environmental factors were analyzed with R vegan package (v2.6-4; Oksanen et al., 2022). Between sites, distinctions in eukaryotic plankton communities (beta-diversity) were scrutinized by virtue of the ordination called non-metric multidimensional scaling (NMDS) using R vegan package (v2.6-4; Oksanen et al., 2022). In accordance with Spearman’s rank correlations, the relationships between the Bray–Curtis dissimilarity of the plankton community and the geographic distance of sites were resolved with R packages of vegan (v2.6-4; Oksanen et al., 2022), pacman (v0.5.0; Rinker and Kurkiewicz, 2017), geosphere (v1.5-18; Hijmans, 2022), and ggplot2 (v3.4.0; Wickham, 2016). Null model (Modin et al., 2020) was calculated using R packages of vegan (v2.6-4; Oksanen et al., 2022), picante (v1.8.2; Kembel et al., 2010), doParallel (v1.0.17; Corporation and Weston, 2022), and foreach (v1.5.2; Microsoft, 2022) to analyze the biome assembly mechanism of the investigated sites, whereafter the results obtained was spatially visualized by ArcGIS Desktop 10.8 (Esri, United States) in a reference method (Yan et al., 2021).

## 3. Results and discussion

### 3.1. Regional environmental characteristics

A territory of nearly 270 kilometers from southwest to northeast along the coast and 180 kilometers from northwest to southeast along the Pearl River Estuary was covered in this research. On account of its special status as the second largest estuary in China, there were four stations set up along the Pearl River estuary (Figure 1, GB-7 to GB-10). Based on the measured water quality parameters, both EC and salinity present a remarkably increasing gradient, among which the EC increased from 3.3 to 50.7 mS/cm, and the salinity increased from 1.82‰ to 32.17‰, specifically. Other parameters did not change significantly along the Pearl River Estuary. What is more, comprehensive interests have been attracted in the phenomenon of low oxygen in the very region of Pearl River Estuary (Wei et al., 2016; Yu and Gan, 2021; Shen et al., 2022). Taking the value that below 2 mg/L of DO as a dividing standard for hypoxia zone (Breitburg et al., 2018), five of all sites investigated in reached the mentioned standard (GB-3B, GB-5B, GB-8B, GB-9B, and GB-13B, concretely). Especially the point GB-5B where the DO counted 0.23 mg/L, indicating a severe hypoxia.

The detection outcomes of the 18 water quality parameters mentioned above in the coastal area of the Great Bay are drawn in Supplementary Figure S1, and the distinction analyses of the parameters between the surface and bottom water are displayed in Supplementary Table S1. As is illustrated in Supplementary Figure S1 and Supplementary Table S1, considerable measure of the quality parameters tested performs significant difference between sites, especially salinity, chlorophyll a, turbidity, irradiance, nitrate, nitrite among the surface layer sites, and DO, chlorophyll a, turbidity, irradiance, nitrate, nitrite and NH_4_^+^ among the bottom layer sites, all varied by more than one order of magnitude. The differences between groups are analyzed by T-test, and the results indicates that there are significant differences in ORP, T, Depth, DO, pH, turbidity, irradiance, and DOC between the surface and the bottom sites (*p* < 0.05, Supplementary Table S1). The gradient of environmental factors builds a solid basis for analysis of the mechanisms affirming the formation of biodiversity and differences in the biotic community. In addition, EC, salinity, chlorophyll a, nitrate, nitrite, NH_4_^+^, PO_4_^3−^, TC, DIC, and TALK are not significantly different between the surface and bottom layer. The distinction of water quality between the surface and bottom layer was further delved by NMDS analysis (Supplementary Figure S2). What came out demonstrates that the surface and bottom sampling sites can be well separated in the NMDS2 direction based on physical and chemical parameters (stress = 0.09).

The analysis of spearman correlation among spatial and environmental variables reveals regularities as the following. First, latitude has a greater impact on the water quality parameters of the bottom points. According to the topography of the Greater Bay Area studied, the further explanation is that the offshore distance of the sampling points that plays a more impactive role on the water quality parameters of the bottom points. Second, there is an obvious correlation between the turbidity and several other water quality parameters in the surface layer, whereas that is not significant in the bottom layer. Third, no noteworthy correlation of DO at all points is found with other parameters. In addition, the salts measured (nitrate, nitrite, NH_4_^+^, and PO_4_^3−^) has no correlation with other parameters except for PO43-in the surface samples, while in the bottom samples, the other three salts are correlated with some parameters except for NH_4_^+^ (Supplementary Figure S3). The results listed above indicate that physical habitats in the Greater Bay Area vary in both vertical and horizontal dimensions, which is also the groundwork for the differences in biological communities at each individual site and water depth.

### 3.2. Regional alpha-diversity of eukaryotic plankton and related environmental factors

In this eDNA survey, a total of 7,295 OTUs were detected, and 2,307 species were annotated (Supplementary Table S2). Overall, species abundance in the bottom layer (1,817 species) is higher than that in the surface layer (1,459 species; Supplementary Figure S4), while for some sites, the surface layer has higher species abundance, and no obvious pattern is found. The most remarkable difference in the number of species is in GB-13 near land, where 1,074 species are detected in the bottom layer, while only 219 species are found in the surface layer (Supplementary Figure S4), which presumably attributes to territorial influence and the largest depth (14.65 m) of this site among several sites close to land.

**Figure 2:**
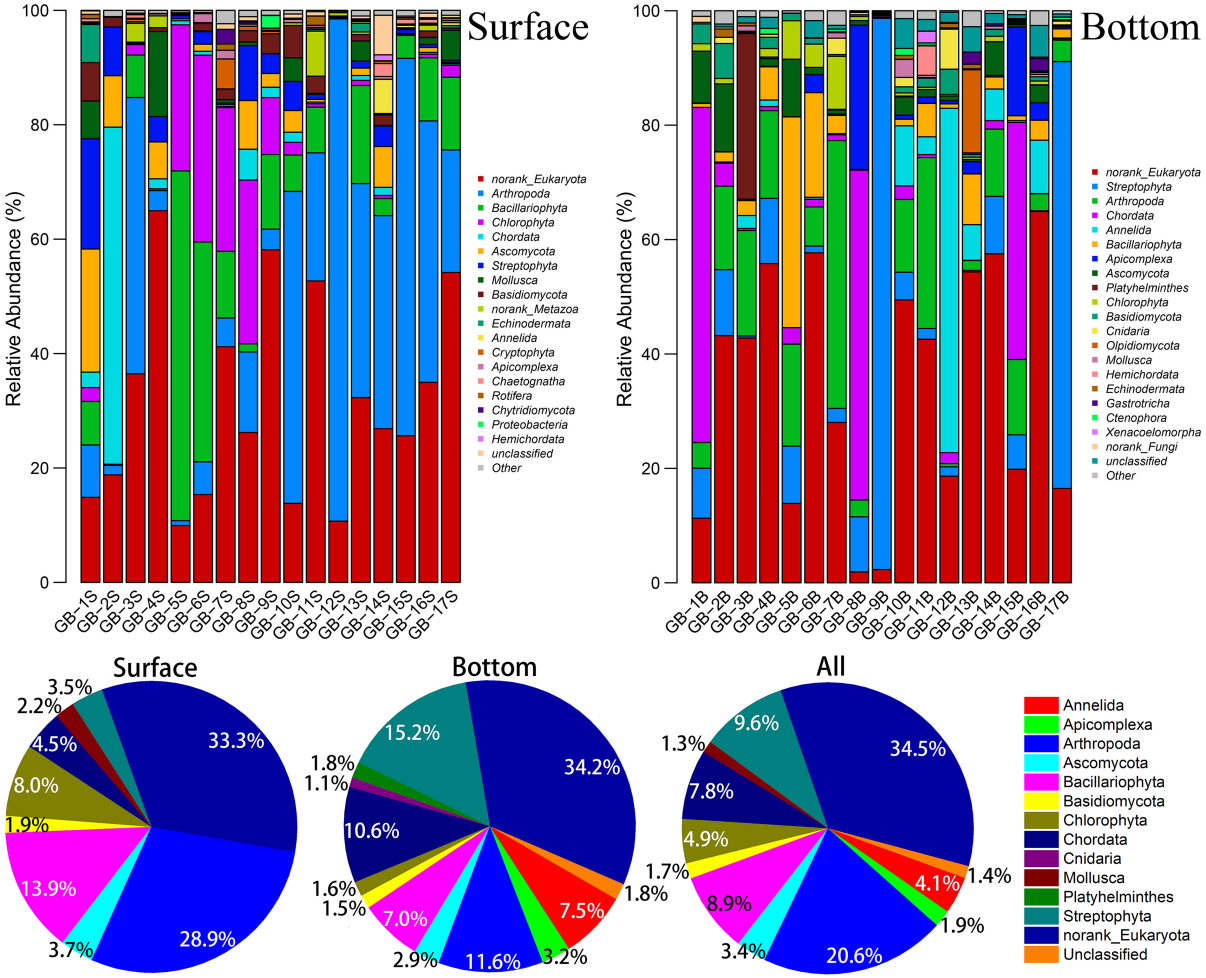
Abundance of dominant eukaryotic plankton at phylum level. The dominant species in the bar chart were those whose OTU number in each site were more than 1%; The dominant species in the pie chart are those whose OTU are more than 1% in the surface, bottom, or both.

The group and abundance of dominant species are shown in Figure 2. According to the number of OTU at each site, Arthropoda is verified to be the major group in the surface waters in the east of the Pearl River Estuary (GB-10S–GB-17S). Species composition in the surface waters in the west of the Pearl River Estuary (GB-1S–GB-9S) varies exceedingly, which primarily are Bacillariophyta and Chlorophyta, whereas some other groups (e.g., Chordata or Arthropoda) appear in a few individual points (GB-2S and GB-3S). In general, Arthropoda and Bacillariophyta accounts for 28.9 and 13.9%, respectively, which are the top two dominant taxa in the surface waters. For the bottom waters, the distribution of dominant species takes on a more diversified characteristic. For instance, for GB-9B and GB-13B, Streptophyta takes an absolute advantage. For GB-1B, GB-8B, and GB-15B, Chordata is dominant. Overall, Streptophyta, Arthropoda, and Chordata are the top three taxa in the bottom waters, accounting for 15.2, 11.6, and 10.6%, respectively. Arthropoda is the largest group in the whole area (combined with surface and bottom data), accounting for 20.6%, followed by Streptophyta, Bacillariophyta, and Chordata, accounting for 9.6, 8.9, and 7.8%. Despite that the diversity of marine plankton has investigated in earlier studies, limited reports have focused on the diversity of near-shore plankton yet. In LÓpez-Garcia’s study of Mid-Atlantic Ridge region on biomes, the dominant position of Alveolata in the deep ocean was found (Lopez-Garcia et al., 2003). Stoeck et al. (2010) found that the two most diverse groups were Dinoflagellates and Chlorophytes in marine anoxic water environment (20 m depth) by eDNA technology. A recently published study on microeukaryotic biodiversity in the northern South China Sea shows that Alveolata and Opisthokonta overwhelmingly dominate the assemblages in the abundance (44.76 and 31.08%) and species richness (59 and 12%; Li et al., 2023). Needham et al. found that typically Ostreococcus, Braarudosphaera, Teleaulax, and Synechococcus dominated phytoplankton sequences in the surface ocean off Catalina Island, California (Needham et al., 2018). The results mentioned earlier about marine eukaryotic diversity have a noteworthy variance with what is unmasked in this study of the Great Bay near shore area, which brings the diversity of biological communities in different sea areas to light.

**Figure 3:**
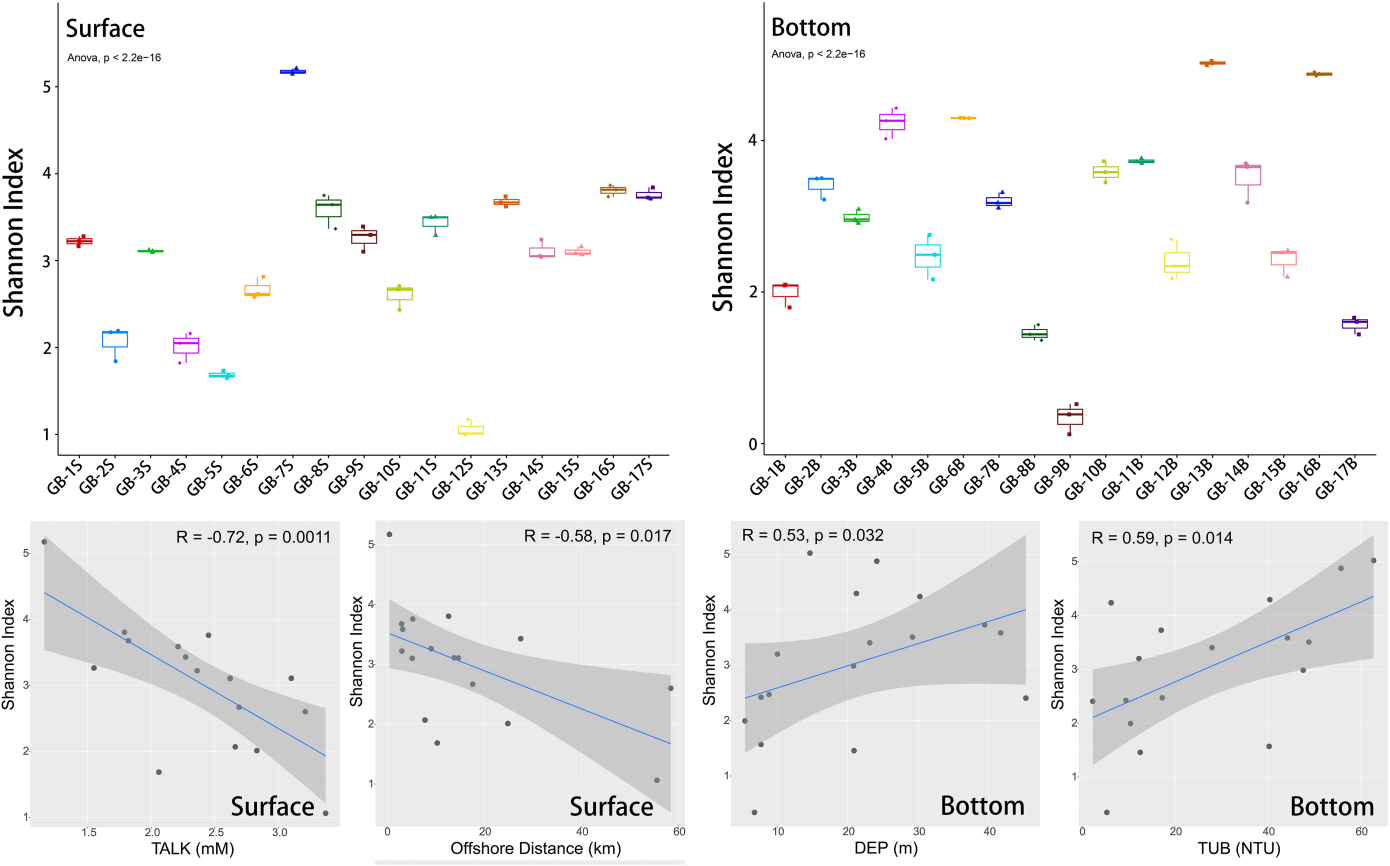
Alpha-diversity (Shannon index) of eukaryotic plankton in surface and bottom layers, and Spearman correlations between Shannon index and environmental factors. In each boxplot, from bottom to top, there are lower limit (minimum value), lower quartile (Q1), median value (Q2), upper quartile (Q3) and upper limit (maximum value). ANOVA test was used for comparison between groups.

Both the alpha-diversity portrayed by Shannon index and its correlation analysis with environmental factors are presented in Figure 3. Overall, the Shannon index of each site ranges from 0 to 5, but there are significant differences among sites (ANOVA analysis, *p* < 2.2e-16). The surface site with the highest diversity is GB-7S (Shannon index = 5.17, located in the deepest estuary), and the surface site with the lowest diversity is GB-12S (Shannon index = 1.06, located furthest offshore). The difference of Shannon index between the two sites varies nearly 5 times. The sites with the highest and lowest diversity in the bottom layer are GB-13B (Shannon index = 5.02) and GB-9B (Shannon index = 0.34), respectively, with a difference of more than 10 times, denoting that the alpha-diversity difference among bottom sites is more pronounced than that among surface sites.

The correlation analysis with environmental factors shows that the factors affecting alpha-diversity in the surface and bottom layers are different. TALK (*p* = 0.0011) and offshore distance (*p* = 0.017) are significantly correlated with Shannon index of the surface layer. On the contrary, for the bottom layer, the depth (*p* = 0.032) and turbidity (*p* = 0.014) are significantly correlated with Shannon index. Meanwhile, more than 10 other environmental factors have no significant correlation with Shannon index of surface or bottom layer (Supplementary Figures S5, S6). Since the coastline of the Great Bay Area is more oriented in the east–west direction, the latitude of the point is closely related to the offshore distance (Supplementary Figure S3). Besides, latitude also has a strong impact on surface biodiversity (*p* = 0.00057, Supplementary Figure S5), following the rule that the greater the latitude, the closer the shore, the higher the biodiversity, which is speculated to be related to the nearshore nutrient discharge and other factors. Moreover, there is no significant correlation between water depth and turbidity at the bottom points (Supplementary Figure S3), yet both are positively correlated with biodiversity at the bottom. The impact of environmental factors on aquatic biodiversity has been widely concerned in previous studies. For example, in Taiwan Strait, the chlorophyll a concentrations, DO concentrations, latitude and bacterial abundance has a prominent impact on alpha-diversity of ciliate (Sun et al., 2020). Benthic community variations has been clarified to be closely related to factors such as the water content of sediment and the concentration of Cd (Kong et al., 2019), and DO concentration (Shin et al., 2014) as well. To sum up in stages, the above studies illuminate that the influence of environmental conditions plays a vital role in the succession of biological communities, and human activities act a considerable impact on coastal biodiversity.

### 3.3. Regional beta-diversity of eukaryotic plankton

**Figure 4:**
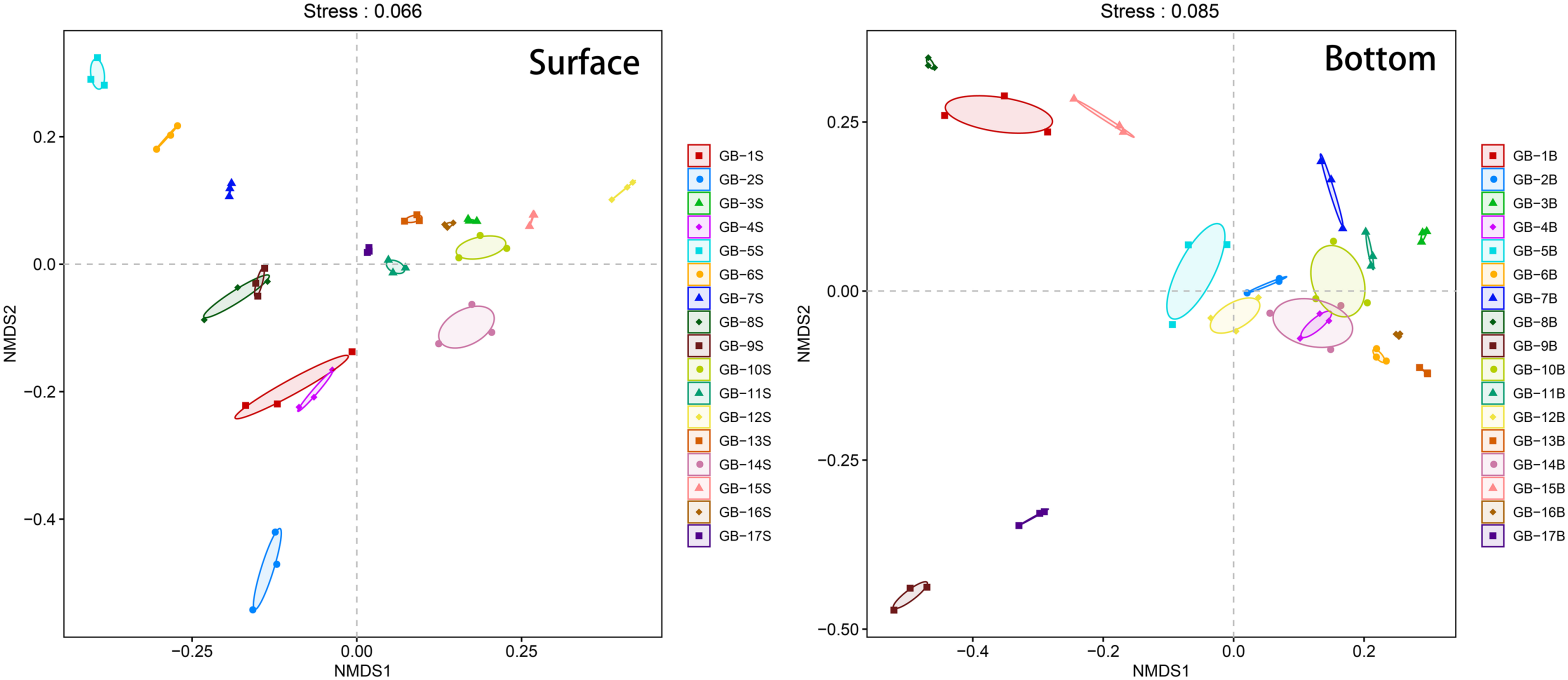
Beta-diversity (Non-metric multidimensional scaling, NMDS) of eukaryotic plankton in surface and bottom layers. The three points corresponding to each site in the figure are three PCR replicates. The Stress parameter is below 0.1, indicating that the sorting results are reliable.

Beta-diversity of the plankton is analyzed using NMDS, and the results are displayed in Figure 4. Conducting a joint analysis of Figure 1 and Figure 4, for surface points, estuary is the key boundaries. The estuary and most points to the west of the estuary (i.e., GB-1S, GB-2S, GB-4S, GB-5S, GB-6S, GB-7S, GB-8S, and GB-9S) are clustered in the negative region in the NMDS1 dimension, and the points to the east of the estuary are clustered in the positive region of the NMDS dimension (Figure. 4). According to what has been studied before (Ji et al., 2011), the direction of surface current in the Pearl River Estuary is affected by tidal current, Pearl River runoff, and earth bias force. The Pearl River Estuary flows westward after entering the ocean, which interprets the reason why the community structure of the Pearl River Estuary point is relatively analogous to that of the western point. For the bottom points, most of them near the land (i.e., GB-1B, GB-5B, GB-8B, GB-9B, GB-15B, and GB-17B) are clustered in the negative region in the NMDS1 dimension, and correspondingly, most of the points far from the land are clustered in the positive region in the NMDS1 dimension. This is also in harmony with the effect of offshore distance on alpha-diversity (Figure. 3), indicating that seawater depth and terrestrial input may have significant effects on the near-shore biomes. Water depth is one of the important factors affecting marine biological communities. Many previous studies have confirmed this point (Amaral-Zettler et al., 2009; USEPA, 2013; Wei et al., 2016).

### 3.4. Environmental factors influencing the eukaryotic plankton

**Figure 5:**
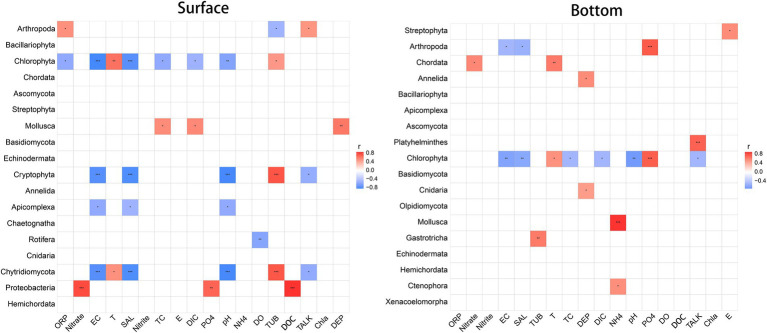
Spearman correlation between biological community and environmental variables. Red indicates positive correlation, while blue indicates negative correlation. *p < 0.05, **p < 0.01, ***p < 0.001.

Correlation analysis between dominant biological communities and environmental factors (Figure. 5) reveals that environmental factors associated to the surface and the bottom biological groups are extremely separate. Also, it is obvious that the surface community is more vigorously correlated with environmental factors, and the paramount related factors (affecting at least four taxa) include EC, salinity, pH, and turbidity. Comparatively speaking, the correlation between bottom layer groups and environmental factors, with no more than two groups affected by any factor, is relatively weaker, and environmental factors that highly correlated with individual biological groups (*p* < 0.001) include NH_4_^+^, PO_4_^3−^, and TALK. It is particularly worth mentioning that because mollusks (larvae) are found so sensitive to ammonia that the original freshwater criteria of ammonia are not enough to protect shellfish, the United States Environmental Protection Agency (USEPA) revised the national ammonia criteria in 2013 (Yan et al., 2020). This finding has also been confirmed in China’s national water quality criteria of ammonia (Kong et al., 2022), which is in obedience to the highly significant negative correlation between marine shellfish and NH_4_^+^ found here. Surprisingly, there is no significant correlation between DO concentrations and the underlying biome by correlation analysis. Whether the several underlying hypoxia stations involved in this study have an impact on the biome still worth further studies.

From the perspective of biological population, Chlorophyta, Cryptophyta, and Chytridiomycota in the surface water are more affected by environmental factors, proved by that at least one third of the 18 environmental factors determined affect them. For the bottom communities, conversely, only Chlorophyta is affected by almost half of environmental factors while the number of factors that are influential to the other communities are much less. For both the surface and bottom layers, barely half of biological communities have no significant correlation with environmental factors.

**Figure 6:**
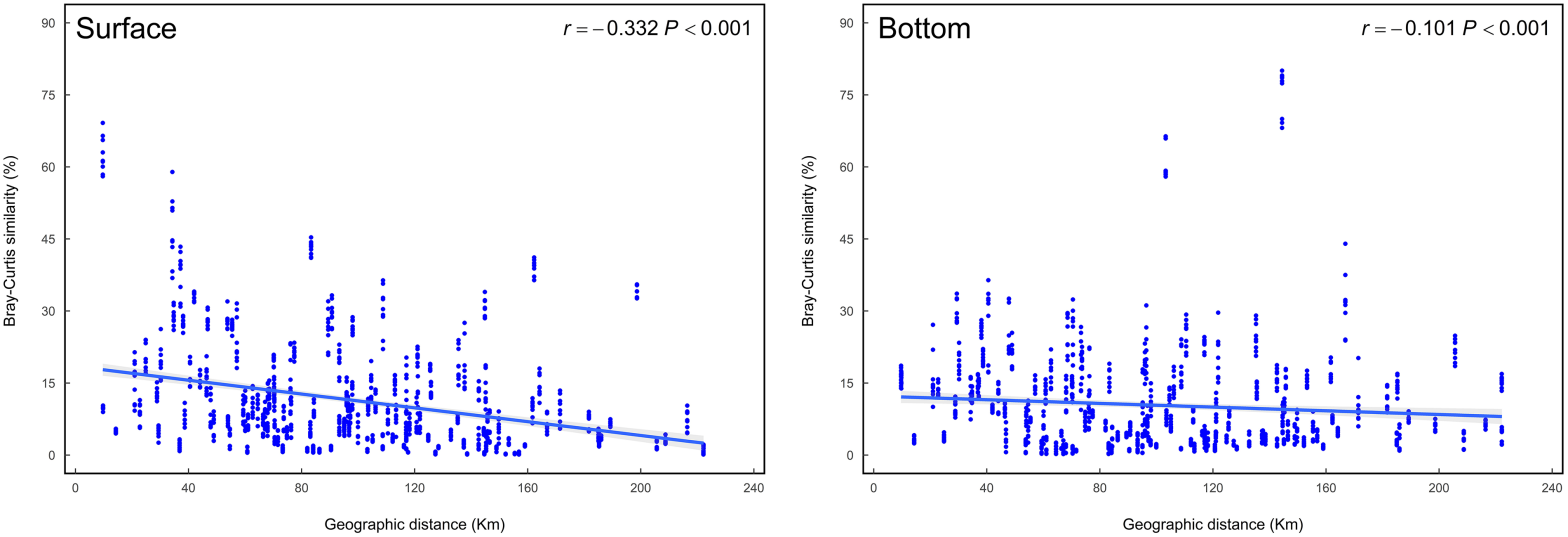
Distance-decay curves showing Bray–Curtis similarity among eukaryotic plankton communities against geographic distances among sampling sites.

It is well-known that geographic distance has an appreciable impact on the differentiation of biomes, and distance-decay pattern is very common to explain the formation mechanism of microbiomes (USEPA, 2013). Isabwe’s research suggested that both bacterioplankton and eukaryotic plankton communities from a subtropical river-reservoir system exhibited significant distance-decay relationships (Isabwe et al., 2019). Wu et al. found that differences between bacteria and protists communities in different sites also increased with increasing geographical distance (Wu et al., 2018). This study demonstrates that as the geographical distance increases, the differences of the surface and bottom communities in the coastal waters tend to increase as well, and the increasing trend is more obvious in the surface region (Figure 6).

### 3.5. Assembly mechanisms of eukaryotic plankton communities

**Figure 7:**
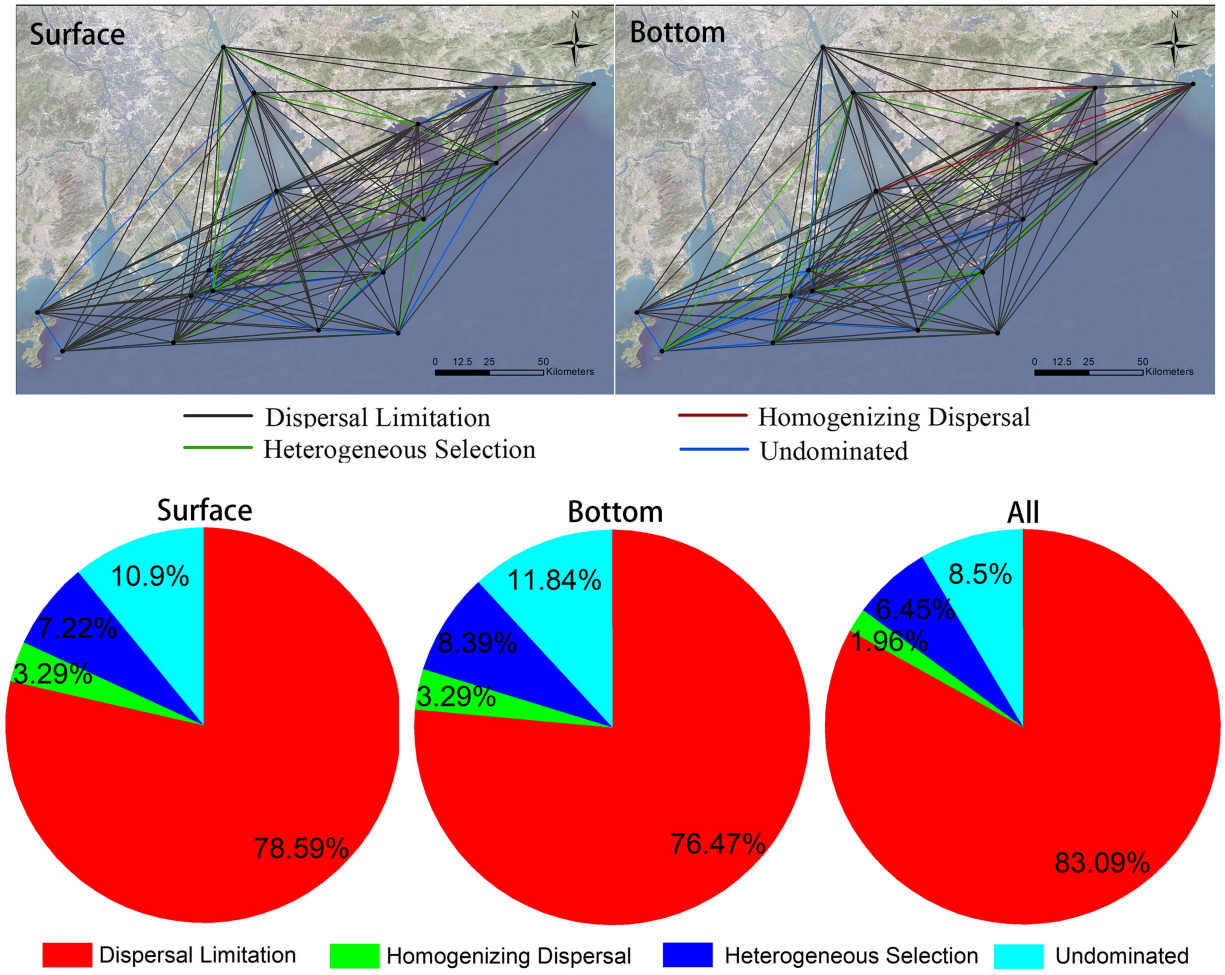
Eukaryotic plankton community assembly mechanisms in coastal waters of the Greater Bay Area, illustrating leading rule of stochastic processes (dispersal limitation).

The mechanism of community assembly is a hot research topic of concern. In this research, we calculate the assembly mechanisms of eukaryotic plankton communities in coastal waters of the Great Bay Area by using null model. The result suggests that the differences in assembly mechanism of community varies in different sites in the surface and the bottom layer (Figure 7, top graph), but either the surface or the bottom communities are composed of dispersal limitation, heterogeneous selection, homogenizing dispersal, and other undominated types. The proportion of each mechanism type in the surface and bottom layer is similar (Figure 7, bottom), indicating that the relative importance of each ecological processes in the two layers is similar. Dispersedly, dispersal limitation is major way that eukaryotic plankton communities’ assembly, accounting for more than 83%, following heterogeneous selection and homogenizing dispersal account for 6.45 and 1.96%, respectively (Figure 7, bottom). In conclusion, stochastic processes are major mechanisms responsible for formation of offshore eukaryotic plankton communities in the Greater Bay Area.

Many studies have been conducted on the assembly mechanism of microbial community previously. It has been found that the relative importance of different assembly processes differs among taxonomic groups and habitats (Langenheder and Lindstrom, 2019; Logares et al., 2020). Logares et al. (2018), for example, reached to a conclusion that habitat diversification could contribute to contrasting assembly mechanisms. In the lakes of East Antarctica, bacterial communities were configured mainly by selection while microeukaryotic communities were structured mainly by ecological drift. When it comes to the tropical and subtropical surface ocean, multifarious mechanisms have participated in the shaping of biogeographies of prokaryotes and picoeukaryotes. Primarily, picoeukaryotic communities are portrayed by dispersal limitation, whereas dispersal, selection, and ecological drift collectively structure prokaryotic communities (Logares et al., 2020). In a study of the Tropical North Pacific Ocean, a view that heterogeneous selection, dispersal limitation, and ecological drift collectively explained much of the turnover of surface microeukaryote communities was presented (USEPA, 2013). Limited research has been taken on the assembly mechanism of marine eukaryotic plankton community. Comparatively, there are certain differences with the results of the plankton community in the nearshore water bodies in this study. In addition to the different species groups studied, the differences between this study and the above results are also related to the marine environment. The above study area is the oceanic area which is vitally different from the coastal area in this study. Moreover, in a study of the subtropical river-reservoir system, of pairwise comparisons, it is the dispersal limitation that accounts for the largest percentage (42–68%) in line accordance with the phylogenetic null model, followed by environmental selection (18–25%; Isabwe et al., 2019). It is similar to our conclusion, but dispersal limitation plays a more crucial role in this study.

## 4. Conclusion

In this study, eDNA was investigated on the eukaryotic plankton communities at 17 sites on both surface and bottom in the coastal waters of the Greater Bay Area, China, gaining 7,295 OTUs from 2,307 species. It is concluded that there are significant differences between surface and bottom biomes. Arthropoda and Bacillariophyta are dominant in surface, while Streptophyta, Arthropoda, and Chordata are dominant in bottom. Broadly speaking, biodiversity of bottom layer is higher than that of surface layer, and differences of alpha-diversity (Shannon index) among different sites in bottom layer is 10 times higher than that in surface layer. Ulteriorly, the environmental factors affecting alpha-diversity in surface and bottom layers are different, too. More precisely, biodiversity of communities in surface are mainly affected by TALK and offshore distance, while in the bottom, are the depth and turbidity. Seawater depth and terrestrial input may contribute much on beta-diversity of coastal communities. Additionally, the biomes investigated in this study also show a typical distance-decay pattern, and the distance decay effect of surface layer is stronger than that of bottom layer. The assembly mechanisms of surface and bottom biological communities are likely to each other and are both dominated by stochastic processes. As for the whole region, dispersal limitation, accounting for more than 83%, is the major way of assembly mechanisms of eukaryotic plankton communities, followed by heterogeneous selection and homogenizing dispersal, which account for 6.45 and 1.96%, respectively.

## Data availability statement

The datasets presented in this study can be found in online repositories. The names of the repository/repositories and accession number(s) can be found in the article/Supplementary material.

## Author contributions

Z-GY contributed to the experimental design, data analysis, and the manuscript draft. X-MZ contributed to the experimental design and data analysis. S-WZ carried out the chemical analysis and data analysis. HJ contributed to the sampling and manuscript writing. S-PW contributed to data analysis. CW, JW, YS, and CL contributed to the sampling. HW contributed to the final proofreading. All authors contributed to the article and approved the submitted version.

## Funding

This study was financially supported by the National Key Research and Development Program of China (nos. 2021YFC3201005 and 2021YFC3101700), and the project of Southern Marine Science and Engineering Guangdong Laboratory (Zhuhai) (no. SML2020SP008).

## Conflict of interest

The authors declare that the research was conducted in the absence of any commercial or financial relationships that could be construed as a potential conflict of interest.

## Publisher’s note

All claims expressed in this article are solely those of the authors and do not necessarily represent those of their affiliated organizations, or those of the publisher, the editors and the reviewers. Any product that may be evaluated in this article, or claim that may be made by its manufacturer, is not guaranteed or endorsed by the publisher.

## Footnotes

^1^ http://www.arb-silva.de/

^2^ http://ncbi.nlm.nih.gov/
