# Supplementary material for: Environmental DNA sequencing reveals the regional difference in diversity and community assembly mechanisms of eukaryotic plankton in coastal waters
Source: Front Microbiol. 2023 Feb 10;14:1132925. doi: 10.3389/fmicb.2023.1132925 (PMC9956185; doi:10.3389/fmicb.2023.1132925)
Supplement: Supplementary file 1 [file Table_1.docx]

**Supplementary Table S1** Difference of water quality parameters between surface and bottom layers

| Parameters | p values (T-test) |
| --- | --- |
| **ORP** | **0.0064** |
| EC | 0.1857 |
| **T** | **0.0001** |
| Salinity | 0.1534 |
| **Depth** | **1.4746E-07** |
| **DO** | **5.516E-05** |
| **pH** | **0.0030** |
| Chl-a | 0.2010 |
| **Turbidity** | **3.7575E-05** |
| **Irradiance** | **0.0015** |
| Nitrate | 0.9462 |
| Nitrite | 0.0979 |
| NH_4_^+^ | 0.3426 |
| PO_4_^3-^ | 0.2668 |
| TC | 0.5171 |
| DIC | 0.2765 |
| **DOC** | **0.0337** |
| TALK | 0.4163 |
