# Supplementary material for: Environmental DNA sequencing reveals the regional difference in diversity and community assembly mechanisms of eukaryotic plankton in coastal waters
Source: Front Microbiol. 2023 Feb 10;14:1132925. doi: 10.3389/fmicb.2023.1132925 (PMC9956185; doi:10.3389/fmicb.2023.1132925)
Supplement: Supplementary file 3 [file Data_Sheet_1.docx]

Supplementary Figures

**
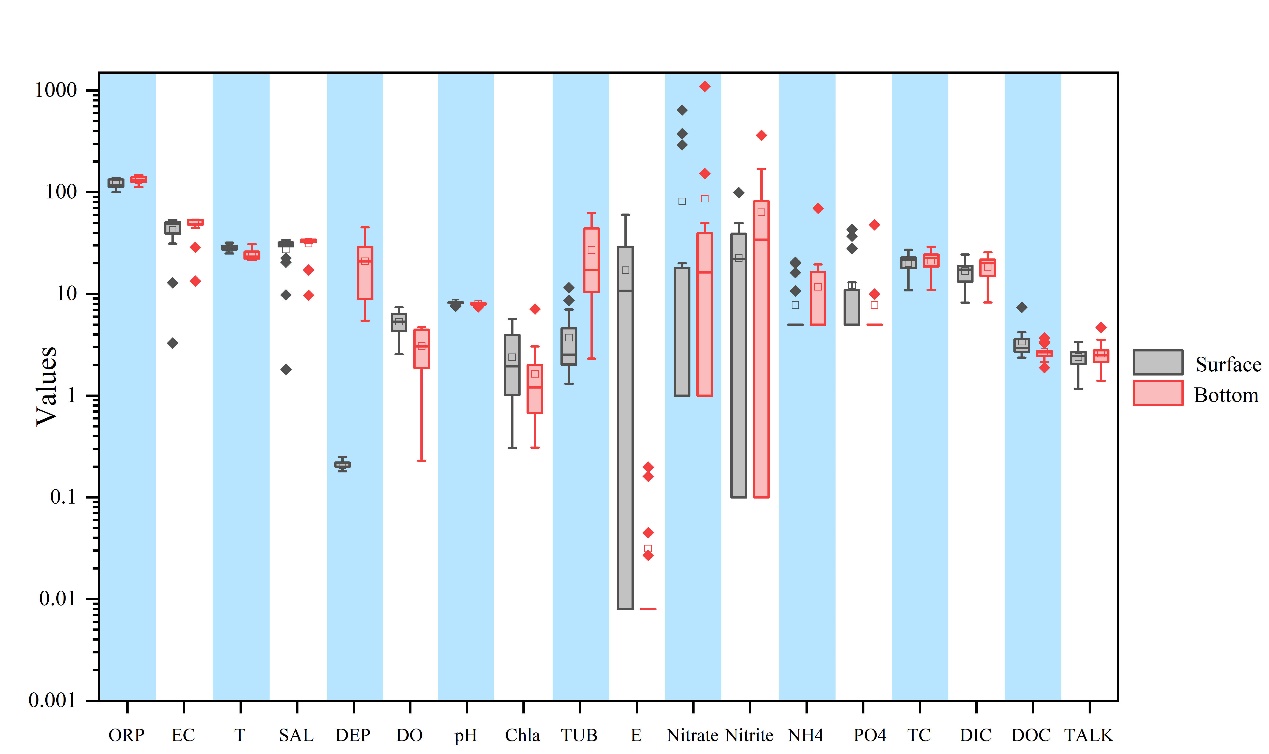
**

**Supplementary Figure S1**  Boxplot of measured water quality parameters in the Greater Bay Area.

**
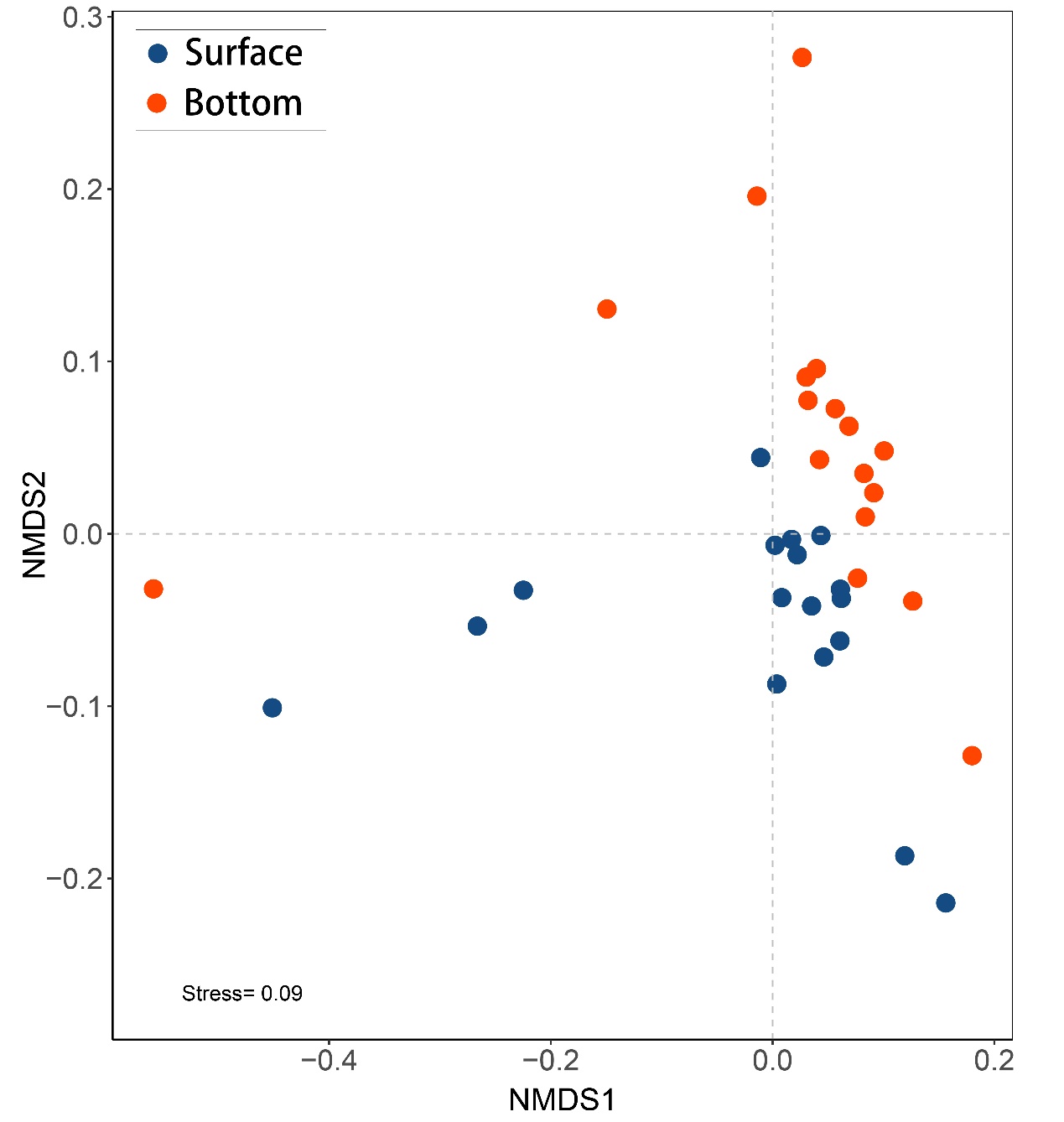
**

**Supplementary Figure S2** NMDS analysis of environmental factors.

**
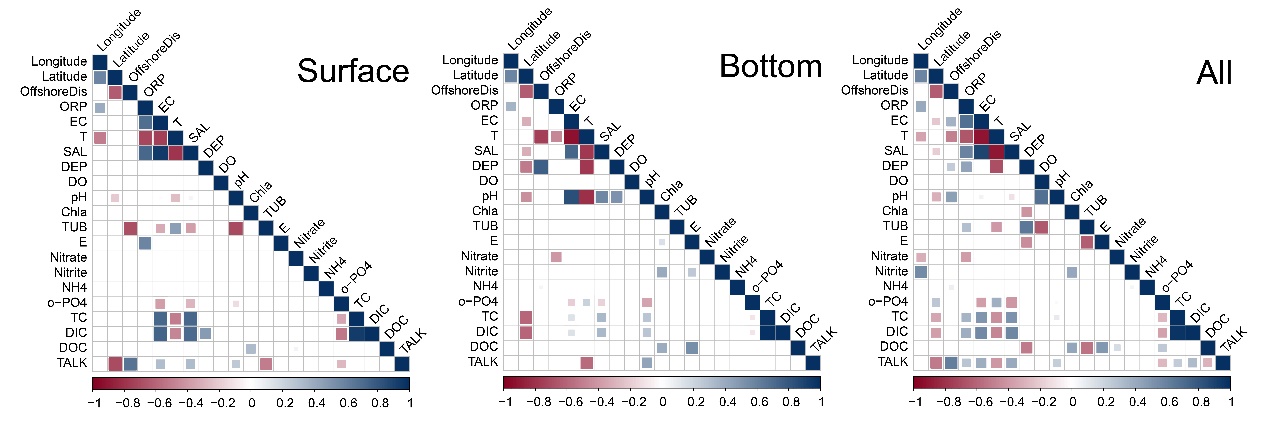
**

**Supplementary Figure S3** Spearman correlation analysis of spatial and environmental factors.

**
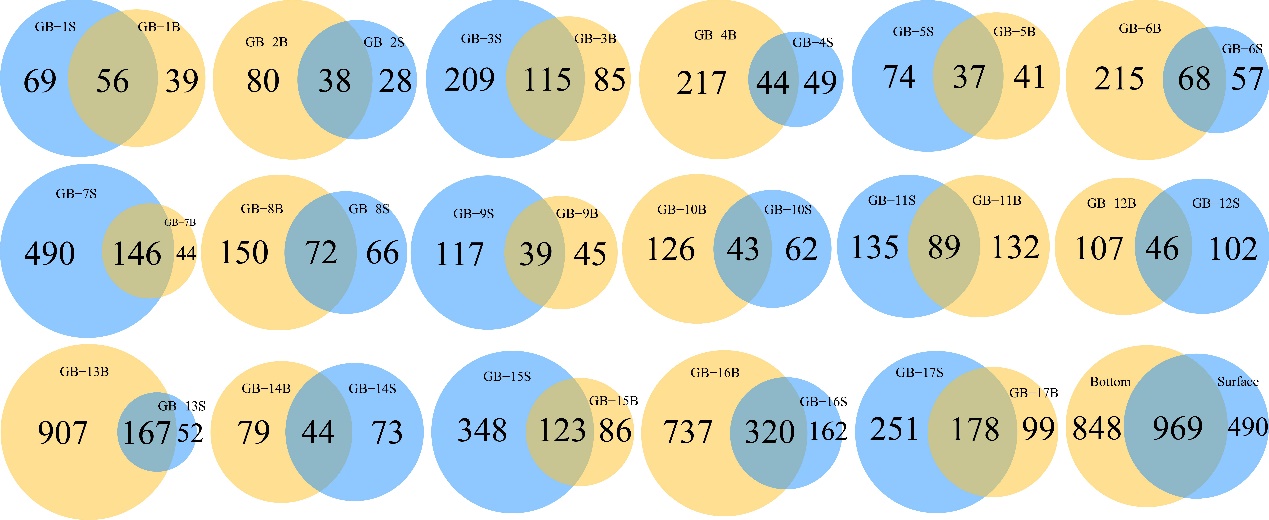
**

**Supplementary Figure S4** Venn diagram of species in surface and bottom sites.

**
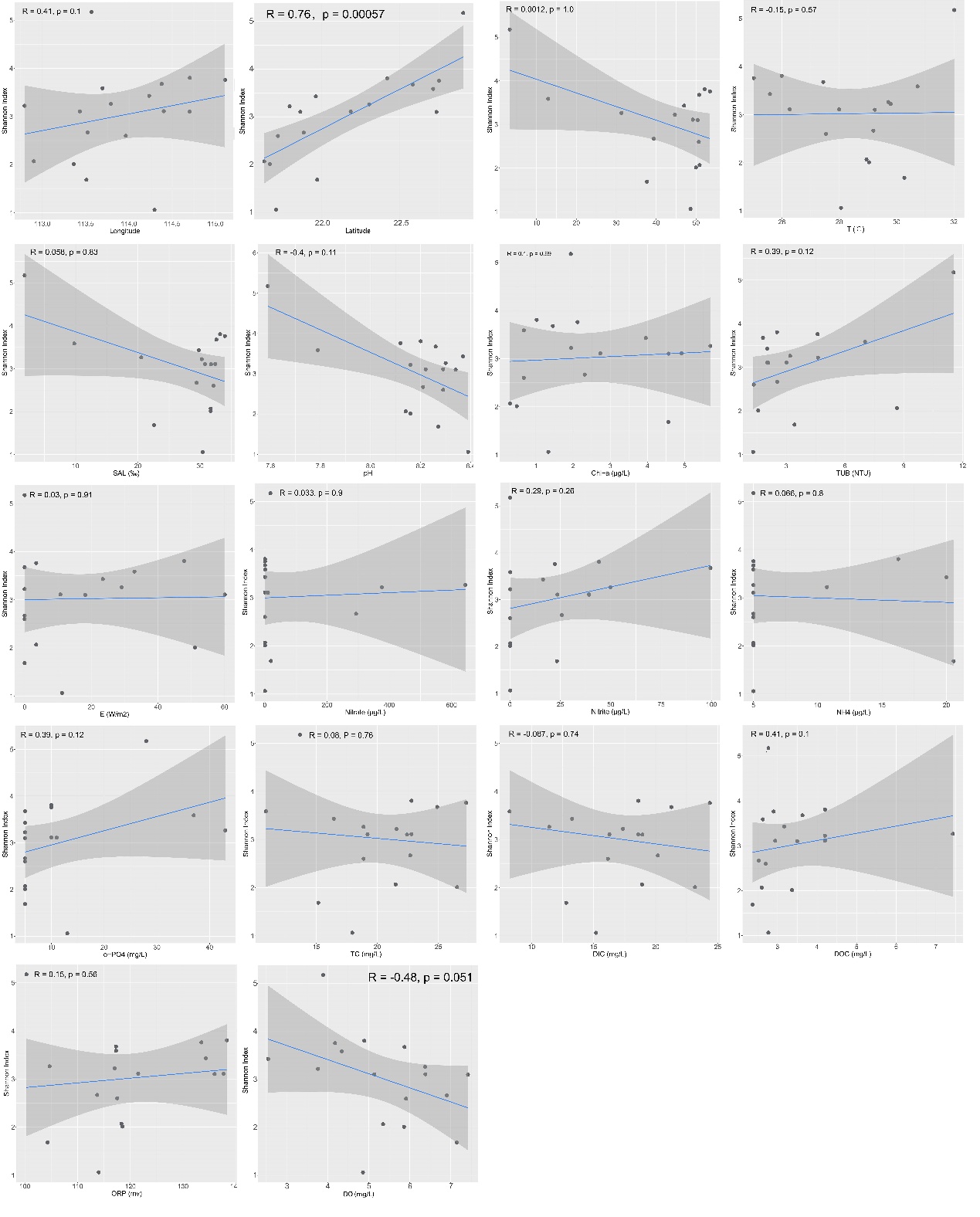
**

**Supplementary Figure S5** Correlation between Shannon index and environmental factors in surface sites.

**
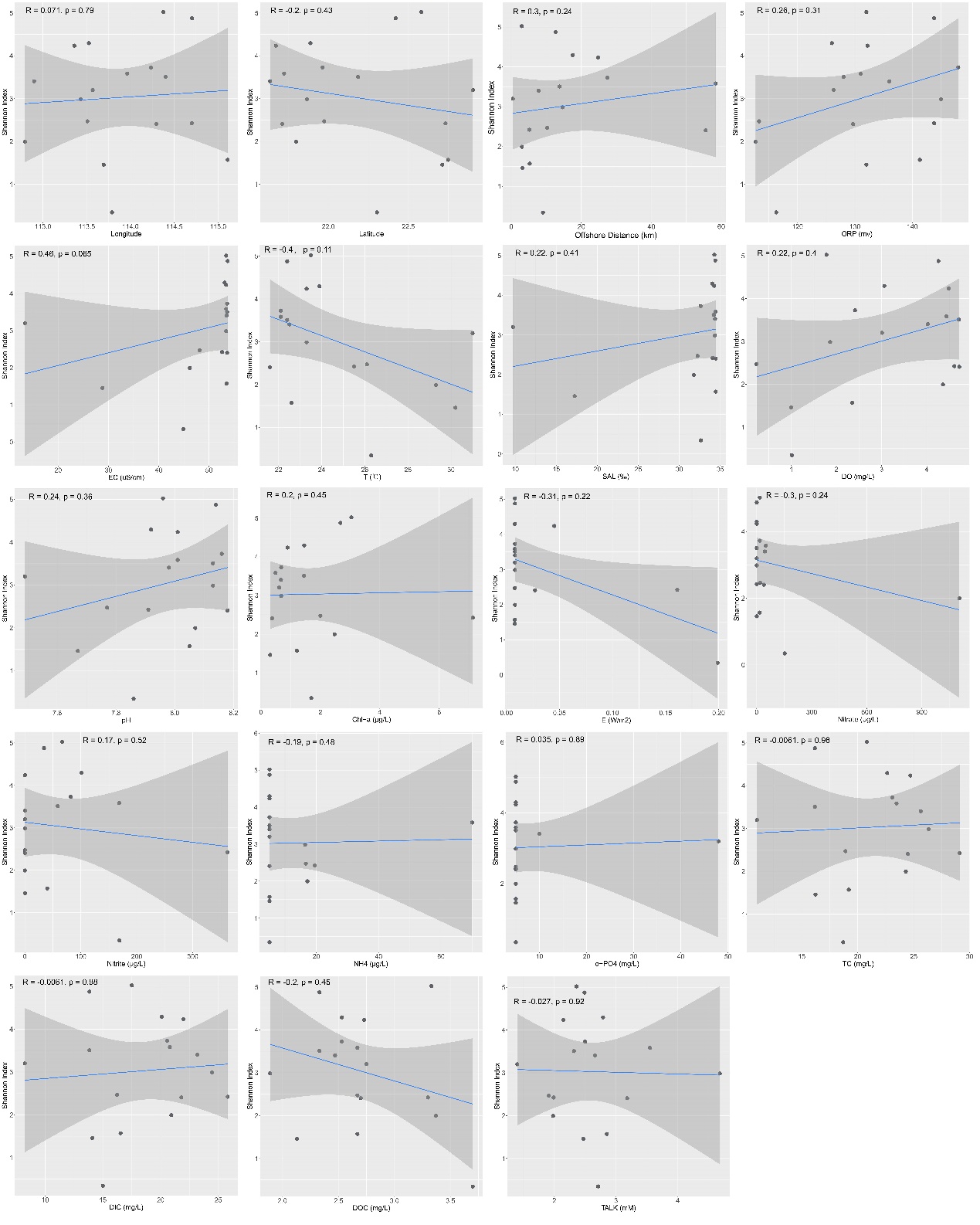
**

**Supplementary Figure S6** Correlation between Shannon index and environmental factors in bottom sites.

Data Availability Statement

The data presented in the study are deposited in the National Center for Biotechnology Information Sequence Read Archive repository, accession number PRJNA901063.
